# Unusually high coral recruitment during the 2016 El Niño in Mo’orea, French Polynesia

**DOI:** 10.1371/journal.pone.0185167

**Published:** 2017-10-10

**Authors:** Peter J. Edmunds

**Affiliations:** Department of Biology, California State University, Northridge, CA, United States of America; Leibniz Centre for Tropical Marine Research, GERMANY

## Abstract

The negative implications of the thermal sensitivity of reef corals became clear with coral bleaching throughout the Caribbean in the 1980’s, and later globally, with the severe El Niño of 1998 and extensive seawater warming in 2005. These events have substantially contributed to declines in coral cover, and therefore the El Niño of 2016 raised concerns over the implications for coral reefs; on the Great Barrier Reef these concerns have been realized. A different outcome developed in Mo’orea, French Polynesia, where in situ seawater temperature from 15 March 2016 to 15 April 2016 was an average of 0.4°C above the upper 95% CI of the decadal mean temperature, and the NOAA Degree Heating Weeks (DHW) metric supported a Level 1 bleaching alert (DHW ≥ 4.0). Starting 1 September 2016 and for the rest of the year (122 d), in situ seawater temperature was an average of 0.4°C above the 95% CI of long-term values, although DHW remained at zero. Minor coral bleaching (0.2–2.6% of the coral) occurred on the outer reef (10-m and 17-m depth) in April 2016, by May 2016 it had intensified to affect 1.3–16.8% of the coral, but by August 2016, only 1.4–3.0% of the coral was bleached. Relative to the previous decade, recruitment of scleractinians to settlement tiles on the outer- (10 m) and back- (2 m) reef over 2016/17 was high, both from January 2016 to August 2016, and from August 2016 to January 2017, with increased relative abundances of pocilloporids on the outer reef, and acroporids in the back reef. The 2016 El Niño created a distinctive signature in seawater temperature for Mo’orea, but it did not cause widespread coral bleaching or mortality, rather, it was associated with high coral recruitment. While the 2016 El Niño has negatively affected other coral reefs in the Indo-Pacific, the coral communities of Mo’orea continue to show signs of resilience, thus cautioning against general statements regarding the effects of the 2015/16 El Niño on coral reefs in the region.

## Introduction

El Niño events arise from the coupling of global atmospheric and oceanographic conditions to perturb atmospheric pressure, winds, rainfall, ocean currents, and sea level throughout the tropical Pacific Ocean [1,2]. These changes result in eastward transport of warm surface seawater to favor warming in central and eastern regions of the equatorial Pacific Ocean [1–3]. Although predicting the occurrence of El Niños remains difficult [3], on average they occur every 3.8 years, with strong events every 9.9 years [4], and their effects can be felt throughout the Indo-Pacific, Atlantic, and Indian Oceans. Negative ecological impacts of these events are common in marine, terrestrial, and freshwater ecosystems [1,5], and they can become catastrophic during strong El Niño events [5,6].

The effects of El Niño often are striking on coral reefs, where they typically are revealed through coral bleaching [1,7,8]. Severe El Niños in 1982–83, 1986–87, and 1997–98, for example, caused large-scale coral bleaching throughout the western Atlantic and Indo-Pacific, and ultimately, widespread coral mortality [1,9–12]. These, and numerous other reports of negative effects of El Niños on coral reefs [8], fueled concerns that coral reefs would be strongly impacted by the severe El Niño that was forecast to develop over 2014–2016 [3,13]. These concerns have been substantiated for the Indo-Pacific, where many reefs experienced severe bleaching beginning in late 2014 and extending into 2017 [14–16], and large areas of some reefs subsequently have died [17].

The primary mechanism by which El Niño impacts reef corals is through warming [18,19], although corals can also be killed by reductions in sea level [1], and intensified storm activity [20]. The absolute increases in seawater temperature attributed to El Niño range from 2–9°C [1,21,22], and these have strong effects on corals [19,23] because they live close to their upper thermal limits [24]. The consequences of high temperature for corals are not straightforward, as the outcome depends on exposure duration, synergy with other stressors, and the temporal structuring of environmental conditions [19,25,26]. Where positive synergy among stressors is strong, corals will bleach, and with persistent bleaching, mortality can be extensive [1,19,26]. The effects of thermal stress on corals can be alleviated through heavy clouds that attenuate synergy with light intensity [27], or the mixing of warm surface seawater with cooler deeper layers by strong storms [28].

As El Niño conditions wane and seawater cools as the event comes to an end, impacted coral communities begin to recover, although the extent and rate of recovery depends on the severity of damage and geographic location of the reef [8,29,30]. Where bleaching is mild and few corals are killed, coral colonies and the communities they build can return to normal coloration in a few weeks [31]. Where bleaching is severe and large numbers of coral colonies are killed, the recovery of the community through replacement of coral colonies can be slow [19], especially when source populations for the coral larvae capable of settling and producing these colonies are distant [32]. El Niño conditions also have effects that are more subtle than bleaching [8], as might be expected from the pervasive influences of temperature on poikilothermic organisms [33]. For scleractinians, these can include impaired reproduction [34,35] that can last more than a year following bleaching [34], probably through the consumption of energy reserves when deprived of carbon from their *Symbiodinium* algal endosymbionts [34,36], and perhaps by shifts in reproductive phenology [37]. The negative impacts of high temperature and bleaching on coral reproduction offer one explanation for reduced coral recruitment following El Niños [22,38,39], as well as shifts in the taxonomic composition of recruiting corals [39–41].

Mo’orea, French Polynesia, provides an interesting location for studying El Niño effects, for the reefs around this island have a well-known ecological history [42–44] that includes multiple El Niño events [43,45]. Interestingly, several coral genera commonly found in the back reef of Mo’orea displayed reduced bleaching incidence across four bleaching events in 1991, 1994, 2002, and 2007, leading to the suggestion that this coral community became less sensitive to thermal bleaching from 1991 to 2007 [46]. Moreover, in the last decade, the outer reefs of Mo’orea have transitioned from high coral cover (e.g., 38% at 10-m depth) in 2005, to minimal cover in 2010 (2%) as a result of corallivory by *Acanthaster planci* and physical damage by Cyclone Oli [47,48], and back to 27% by 2015. Unlike most studies of El Niño effects on coral reefs that address coral cover and bleaching, the present study focused on coral recruitment in Mo’orea to address two questions: (1) to what extent would the 2016 El Niño affect the density and taxonomic composition of coral recruits, and (2) assuming effects would be detected, do they have the potential to promote change in coral community structure? Since the outer reefs in Mo’orea have undergone transition in coral community structure since 2010 [47,48], with recruitment potentially modulated by coral larvae passing between the outer and back reef habitats [49], the results were also used to test for the effects of larvae connectivity between the outer- and back- reef in terms of spatial variation along the reef in density and taxonomic composition of coral recruits.

## Materials and methods

### Overview

The study was conducted on the north shore of Mo’orea, where coral reefs have been studied for decades [42–44]. A portion of these data provide a temporal context to the ecological trends characterizing the reefs in 2016 in terms of seawater temperature, coral bleaching, and the density of coral recruits on settlement tiles sampling outer- and back- reef habitats. These data were augment with a record of seawater temperature determined from remote sensing through NOAA Coral Reef Watch (CRW), and sampling in 2016 that quantified coral bleaching on the outer reef as well as coral recruitment on the outer- and back- reefs. Bleaching and recruitment were analyzed using methods identical to those employed in the time series analysis.

### Seawater temperature

**Fig 1:**
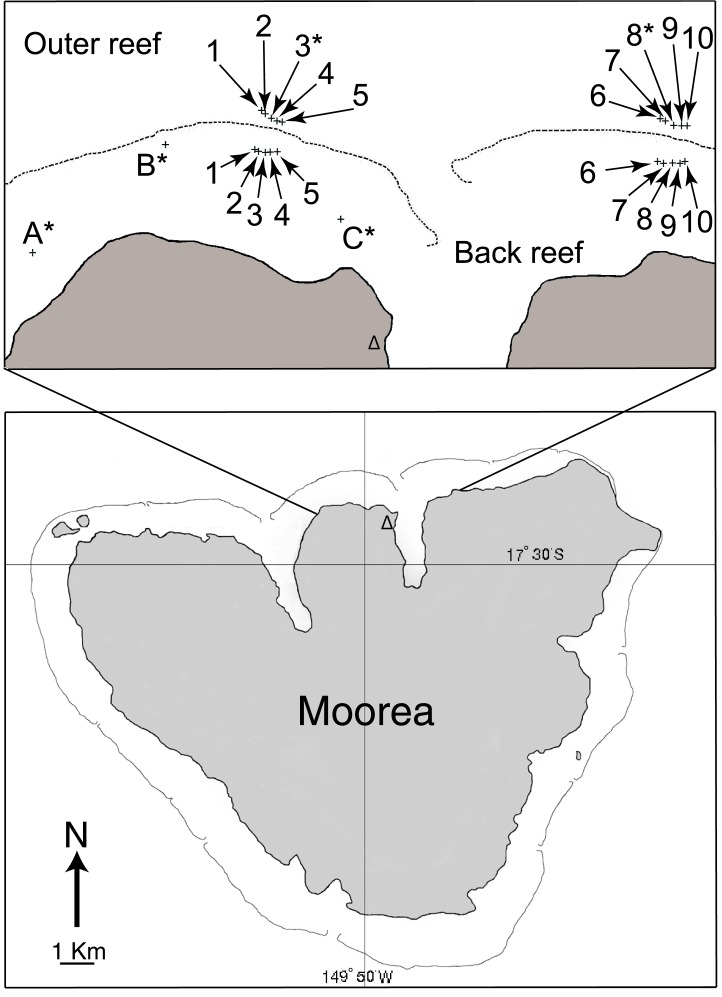
Map of Mo’orea showing Richard B Gump South Pacific Research Station (triangle) and locations of settlement tile deployments (crosses) in the back reef (1–10 and A-C at 1–2-m depth) and outer reef (1–10 at 10-m depth) relative to the long-term study sites (LTER 1 and 2) of the Mo’orea Coral Reef LTER. * = sites at which settlement tiles (n = 15 site^-1^) have been deployed since as early as 2005 and are replaced in about January and August; all other sites had ≈ 10 tiles site^-1^ deployed from January 2016 –August 2016 and August 2016 –January 2017. The outer reef was photographically sampled at 10-m depth at Site 3* and 8* from 2005–2016, and seawater temperature was recorded at 1.5-m depth at Site B*.

In situ seawater temperature was recorded with loggers (Hobo Pro v2, ± 0.2°C, Onset Computer Corp., Bourne, MA) at ≈ 1.5-m depth, ≈ 100 m behind the reef crest (Site B*, Fig 1). Loggers recorded at 0.002 Hz and were installed in September 2005, and exchanged in approximately January and August of each year. Temperature records were averaged by day, and daily values were used to characterize each year, although not all months were sampled due to equipment losses. The thermal environment of seawater on the north shore of Mo’orea was described by the mean and 95% confidence interval (CI) by day between 2005 and 2015. In situ daily seawater temperature in 2016 was interpreted relative to this record. A broader perspective of seawater temperature was provided through NOAA CRW for the “Society Archipelago”, which is a Regional Virtual Station represented by a 5 × 5 km cell centered on 151.375°E 16.950S (https://coralreefwatch.noaa.gov/ accessed 29 August 2017). These data describe nighttime ocean temperature at the surface, and here the 90^th^ percentile hotspot values are reported for the station, together with the degree heating weeks (DHW) calculated from them.

### Coral cover and bleaching

To evaluate the impacts of the 2016 El Niño on the coral community, photoquadrats (0.5 × 0.5 m) recorded as part of the Mo’orea Coral Reef (MCR)–Long-Term Ecological Research site (LTER) time series were quantified for coral cover and bleaching. Photoquadrats from 10-m and 17-m depth on the outer reef at LTER 1 and LTER 2 (Fig 1) were analyzed, with the first images from 4 April 2016 as part of the annual surveys. This project is designed to capture 40 photoquadrats at each depth and site combination every year, although this number sometimes is slightly reduced by logistical constraints associated with data collection. Scheduled sampling of these sites was augmented with sampling to quantify bleaching on 3 May 2016, 10 May 2016, and 27 August 2016. Photoquadrats were recorded with a Nikon D7000 ([16 MP] on 4 April and 27 August) or Nikon D810 ([36 MP] on 3 May and 10 May) camera, fitted with a zoom lens (Nikon DX 18–70 mm or FX 18–35 mm, respectively), and placed in a waterproof housing (Ikellite) that was attached to strobes (Nikon SB105). Photoquadrats were recorded along a permanent transect, with positions determined randomly in 2005, but the same positions resampled thereafter. Photoquadrats were analyzed manually using CoralNet (https://coralnet.ucsd.edu/) with 200 randomly-located points that were identified with a dichotomous label set as either live and normally-colored scleractinians (i.e., healthy), or bleached scleractinians (i.e., white and living corals). For the MCR-LTER project, an expanded label set is applied to resolve corals to genus. Total percentage coral cover was obtained from sum of the healthy and bleached scleractinians, and bleached coral abundance was expressed as a percentage of total coral cover.

### Coral recruitment

Coral recruitment was quantified using unglazed terracotta tiles (15 × 15 × 1 cm) secured individually and horizontally to the benthos with their rough side downward. Tiles were seasoned for 5–6 months in seawater prior to deployment, and when deployment was scheduled, they were removed from the dock several days before installation, lightly cleaned to remove macrofauna, and taken to the reef to be exchanged with tiles that were already in place. As seasoning was completed in a dark and sediment-rich environment beneath the lab dock, and tiles were stacked in piles, their surfaces had few fouling organisms when they were retrieved. Tiles were attached individually to the reef with a stainless steel stud epoxied into rock that held them ≈ 1 cm above the benthos to create a cryptic location favored by settling corals [50]. As part of the MCR-LTER time-series, settlement tiles first were deployed in August 2005 to three back reef sites (≈ 2-m depth), but the sampling was expanded to 10-m depth on the outer reef in 2006, and to 17-m depth on the outer reef in 2007 (Fig 1). Tiles were deployed (and retrieved) twice each year, with deployments sampling from August to January, and January to August, and each consisting of 15 tiles site^-1^; the exact dates of tile deployments differed slightly among years. Sampling was expanded in 2016 to test for an effect of El Niño on coral recruitment, with the expansion consisting of additional tiles deployed at more sites at 10-m depth on the outer reef, and at ≈ 2-m depth in the back reef. Four additional sites were added to the outer reef at LTER1 and 2, flanking the MCR-LTER sites, and spaced ≈ 50-m apart along the isobath. Five back reef sites were added ≈ 150 m behind the reef crest at LTER 1 and 2, and were paired longitudinally with the outer reef sites to test for concordance in recruitment between habitats. Each site was fitted with 10 tiles in January 2016. With the aforementioned sampling, the MCR-LTER time-series sampled ≈ 60 tiles sampling^-1^ on the outer reef, and 45 tiles sampling^-1^ on the back reef, and sampling in 2016 added 80 tiles sampling^-1^ to the outer reef, and 100 tiles sampling^-1^ to the back reef.

Tiles were replaced in late January and late August, with sampling occasionally spilling in to early February and early September, respectively; for clarity, tile recover/exchange is described as occurring in January and August. Freshly collected tiles were placed into dilute domestic bleach to remove organic material, dried, and scored for coral recruits using a dissecting microscope (≈ 40 x magnification). Coral recruits were identified to family (Poritidae, Acroporidae, and Pocilloporidae [51,52]) by a single observer, with all other recruits placed in an “unknown” category. Recruits were scored by location on the top, bottom, and sides of the tiles. Following scoring, carbonate residues were completely removed from the tiles by soaking then in dilute HCl until their surfaces were fully-exposed terracotta. Cleaned tiles were washed in fresh water, and returned to beneath the dock for seasoning in seawater prior to the next deployment. Tiles placed at the four eastern-most of the sites sampled in 2016 in the back- and outer- reef at LTER 1 and 2 were scored alive in August 2016, and returned to the reef within 24 h with the objective of evaluating survivorship of coral recruits at the next sampling. These tiles subsequently were damaged and were omitted from analyses in January 2017, and some of the scores from the initial sampling in August 2016 were excluded due to inaccuracies. The density of recruits summed among the top, bottom, and edges of the tiles (for a total of 510 cm^2^ of area) were expressed as recruits tile^-1^. Typically > 80% of recruits were found on the lower surface of the tiles (e.g., 84% in 2015, n = 198 recruits).

### Statistical analyses

Thermal extremes in 2016 were identified by separation of the daily temperature records from the 95% CI of the long-term temperature records. The percentage of the coral cover bleached (arcsine transformed) was compared among times, within depths and sites using repeated measures (RM) ANOVA. Recruit density was compared among times at each site/depth combination using one way ANOVA, and for the 2016 sampling, the density of recruits was compared among sites and times using two way, Model III ANOVA. Where many tiles often had no recruits (i.e., the back reef) densities were sqrt(x + 3/8) transformed [53]. To test for unusual recruitment densities in 2016, sites were treated as replicates, and mean densities at these sites was compared using t-tests with the mean densities recorded over time in the MCR time-series in the same habitat. Contingency tables with **χ**^2^ tests were used to test for changes in taxonomic composition by family (four columns) of recruits between times (two rows, 2016 versus all other years) for the January-August and August-January samplings. Finally, concordance between recruitment in the outer- and back- reef was tested using Pearson correlations with best-fit linear relationship determined using Model II regressions. Statistical analyses were conducted using Systat 13 software.

## Results

### Seawater temperature

**Fig 2:**
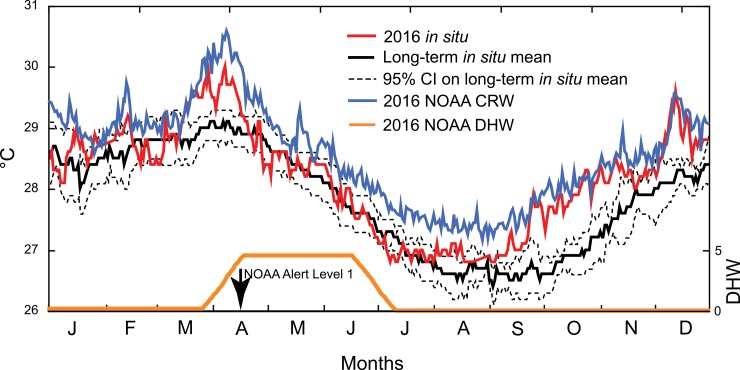
Seawater temperature (°C, left ordinate) around Mo’orea based on remote sensing from NOAA Coral Reef Watch (CRW), and in situ loggers placed at ≈ 1.5-m depth behind the reef crest (Site B* in Fig 1). NOAA CRW data (blue line) are for a 5 × 5 km cell centered on 151.375°E 16.950°S for 2016, and the degree heating weeks (DHW, right ordinate) for this period are shown in orange; on 14 April 2016, NOAA issued a Level 1 Alert for the region based the likelihood of coral bleaching (4 ≤ DHW ≤ 8.0). In situ data have been recorded from 2006 to present (abscissa, months showing January (J), February (F), etc.). Solid black line and dashed black lines show mean daily temperature over 6–9 y, together with their 95% confidence intervals (CI) (respectively); red line shows temperature in 2016.

Based on long-term, in situ records of seawater temperature from 2006 to 2015, seawater flowing over the reef crest at LTER 1 ranged in daily mean temperature from 25.4°C (several days in August 2006) to 29.9°C (several days in March 2006) (Fig 2). In 2016, mean daily temperatures ranged from 26.8°C (several days in July, August and September) to 30.0°C (7 April), which is the local thermal threshold for coral bleaching (www.coralreefwatch.noaa.gov). Based on daily records from 2006 to 2015, the 95%CI on the mean daily temperature was ± 0.3°C, and the daily seawater temperature in 2016 was above this range (i.e., it was significantly warmer) on numerous days. Thermal excursions were striking for 31 days from 16 March, when their mean was 0.4°C (SE < 0.1°C) greater than the upper 95% CI for the long-term temperature record, and for 122 days from 1 September to the end of the year, when their mean was 0.5°C (SE < 0.1°C) greater than the upper 95% CI for the long-term temperature (Fig 2). In situ records of seawater temperature closely corresponded NOAA CRW data for the Society Archipelago virtual station (Fig 2), although nighttime surface temperatures (i.e., CRW data) often were slightly higher than in situ temperatures. Based on CRW data, and the DHW values calculated from them, NOAA issued a Level 1 bleaching alert for 15 April 2016, which corresponded to the mild bleaching observed on the outer reef (described below).

### Coral cover and bleaching

**Fig 3:**
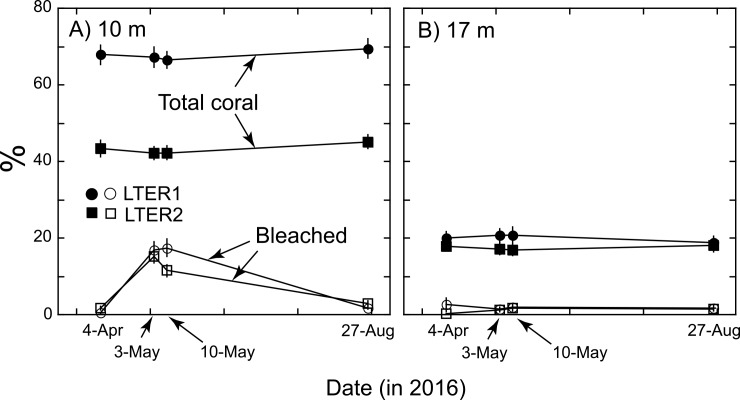
Percentage coral cover (filled symbols) and percentage of corals bleached (open symbols and bars) at 10-m (A) and 17-m (B) depth at LTER1 (3*) and LTER 2 (8*) and four times in 2016 (Fig 1). Mean ± SE shown based on 37–38 photoquadrats at each depth, site, and census; abscissa scaled by number of days.

On 4 April 2016, some of the corals at 10-m depth at LTER1 and 2 appeared pale. At these sites, *Pocillopora meandrina*, *P*. *verrucosa*, and *P*. *eydouxi* (combined) accounted for 74% of the coral cover at 10-m depth, and 68% at 17-m depth (both in 2016), and most (e.g., 56% of 799 colonies at 10 m and 17 m depth at LTER 2) that were categorized as bleached or mottled on 4 April were pocilloporids. The extent of bleaching increased to early May, but three months later (August 2016) bleaching was uncommon. In January 2017, bleaching was not evident on the outer reef, and there were no striking changes in cover of live coral (relative to the previous year). On 4 April 2016, mean coral cover at 10-m depth was 67.9 ± 2.5% at LTER 1, and 43.4 ± 2.1% at LTER2, and at 17-m depth, it was 20.0 ± 1.7% and 17.8 ± 0.2%, respectively (n = 37–38). The percent of live coral that was bleached at 10-m depth was 0.4 ± 0.1% at LTER1 and 1.6 ± 0.5% at LTER2, and at 17-m depth it was 2.6 ± 1.8% at LTER 1 and 0.2 ± 0.1% at LTER 2 (Fig 3). At 10-m depth, the mean proportional cover of bleached coral increased to 11.5–16.8% between 3 and 10 May at both sites, but by 27 August 2016 it had declined to 1.8–3.0%, and there was no reduction in coral cover (Fig 3). Statistical analyses support these trends, with the percentage of coral categorized as bleached at 10-m depth differing among times at both sites (F ≥ 51.316, df = 3,108–111, P ≤ 0.001), while coral cover remained unchanged (F ≤ 2.484, df = 3,108–111, P ≥ 0.064). At 17-m depth, the percentage of coral cover that was bleached remained low, it did not differ among dates at either site (F ≤ 1.868, df = 3,108, P ≥ 0.139), and neither did overall coral cover (F ≤ 0.534, df = 3,108, P ≥ 0.660).

### Coral recruitment

**Fig 4:**
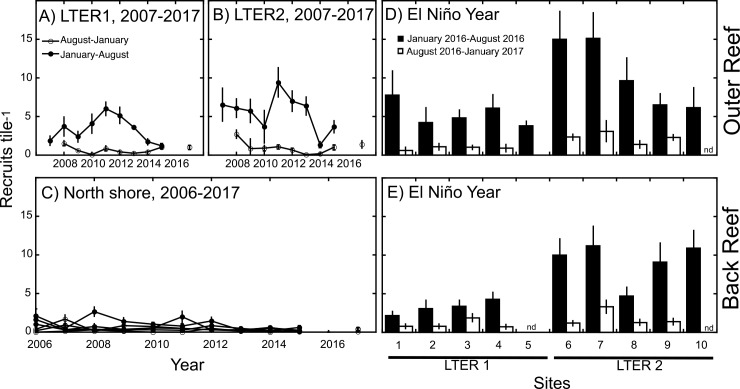
Mean scleractinian recruitment (± SE, n = 7–15 tiles) to settlement tiles deployed horizontally from January-August and January-August at multiple sites (as in Fig 1) and years from as early as 2005. (A,B) Recruitment from 2007–2017 (excluding 2016, shown in D) at 10-m depth on the outer reef at site 3* and 8*, (C) recruitment from 2005–2017 (excluding 2016, shown in E) at ≈ 2-m depth in the back reef at sites A*, B* and C*, (D) recruitment from January 2016-August 2016 and August 2016-January 2017 at 10 outer reef sites (1–10), and (E) recruitment from January 2016-August 2016 and August 2016-January 2017 at 10 back reef sites (1–10). nd = no data.

Sampling of settlement tiles between 2005 and 2015 revealed that coral recruitment was, on average, 5.5-fold greater (n = 18 deployments) on the outer reef (10-m depth) versus the back reef, and mean densities of recruits were 2.3-fold (back reef) and 10.5-fold (outer reef) greater over January-August versus August-January (Fig 4A–4C). On the outer reef, coral recruitment between January and August differed among years from 2007–2015 at LTER 1 (F = 2.363, df = 8,125, P = 0.021) and LTER 2 (F = 2,438, df = 8,128, P = 0.017), and at LTER 1 it ranged from 1.20 ± 0.30 recruits tile^-1^ (2015) to 6.00 ± 0.89 recruits tile^-1^ (2011), and at LTER 2, from 1.33 ± 0.39 recruits tile^-1^ (2014) to 9.41 ± 1.90 recruits tile^-1^ (2011). Between August and January, recruitment also differed among years at LTER 1 (F = 3.369, df = 7,112, P = 0.001) and LTER 2 (F = 7.380, df = 7,113, P < 0.001), with mean densities of 0.64 ± 0.17 recruits tile^-1^ (LTER 1) and 1.22 ± 0.65 recruits tile^-1^ (LTER 2) (± SE, n = 8 y). In the back reef (Sites A*–C*) recruitment was lower, but still differed among years from 2006–2015 between January and August at all sites (F ≥ 2.068, df = 8,118–131, P ≤ 0.044), and between August and January at sites C* and B* (F ≥ 2.012, df = 9,133–140, P ≤ 0.042), but not A* (F = 1.640, df = 9,140, P = 0.109). Between August and January mean recruitment ranged from 0.12 ± 0.03 recruits tile^-1^ (Site C*) to 0.16 ± 0.03 recruits tile^-1^ (Site B*), and between January and August mean recruitment ranged from 0.24 ± 0.06 recruits tile^-1^ (Site A*) to 0.32 ± 0.06 recruits tile^-1^ (Site B*) (all ± SE, n = 11–12 y).

Recruitment in 2016 differed from recruitment in the preceding decade on the outer (10 m) and back (Fig 4) reef. On the outer reef (Fig 4D), the highest mean recruitment in 2016 at LTER 1 (7.78 recruits tile^-1^, Site 1) and LTER 2 (15.1 recruits tile^-1^, Site 7) was higher than recorded over the previous decade at both sites. Between January and August for the outer reef sites, the lowest mean recruitment rates at LTER 1 (3.78 recruits tile^-1^, Site 5) and LTER 2 (6.13 recruits tile^-1^, Site 10) were higher than recorded at the respective sites in 6 of 10 years at LTER1, and 5 of 10 years at LTER 2. Between August and January, recruitment on the outer reef mostly was higher than that recorded over the previous decade, with the highest mean recruitment in 2016 at LTER 1 (1.10 recruits tile^-1^, Site 3) and LTER 2 (2.33 recruits tile^-1^, Site 2) both higher than recorded at their respective sites in 9 of 10 preceding years at LTER 1 and 2. Overall, mean recruitment (± SE) at 10-m depth on the outer reef between January and August 2016 (7.90 ± 1.31 recruits tile^-1^ [n = 10 sites]) was 1.78-fold greater than recorded over the previous decade (4.42 ± 0.53 recruits tile^-1^ [n = 18 records]) (t = 2.901, df = 26, P = 0.007). Likewise, mean recruitment between August 2016 and January 2017 (1.59 ± 0.31 recruits tile^-1^ [n = 8 sites]) was 2.2-fold greater than over the previous decade (0.79 ± 0.16 recruits tile^-1^ [n = 16 records]) (t = 2.505, df = 222, P = 0.020).

Similar trends were recorded in the back reef during 2016 (Fig 4E), where the highest mean recruitment between January and August (11.20 recruits tile^-1^, Site 7) and August and January (3.3 recruits tile^-1^, Site 7) exceeded recruitment in the back reef over the previous decade (Fig 4C and 4E). Between January and August 2016, mean recruitment (± SE) (6.54 ± 1.23 recruits tile^-1^ [n = 9]) was 9.0-fold higher than over the previous decade in the same period (0.73 ± 0.15 recruits tile^-1^ [n = 30]) (t = 8.463, df = 37, P < 0.001). Likewise, mean recruitment (± SE) between August 2016 and January 2017 (1.43 ± 0.30 recruits tile^-1^ [n = 8]) was 4.0-fold higher than over the previous decade in the same period (0.36 ± 0.08 recruits tile^-1^ [n = 30]) (t = 5.473, df = 36, P < 0.001).

**Fig 5:**
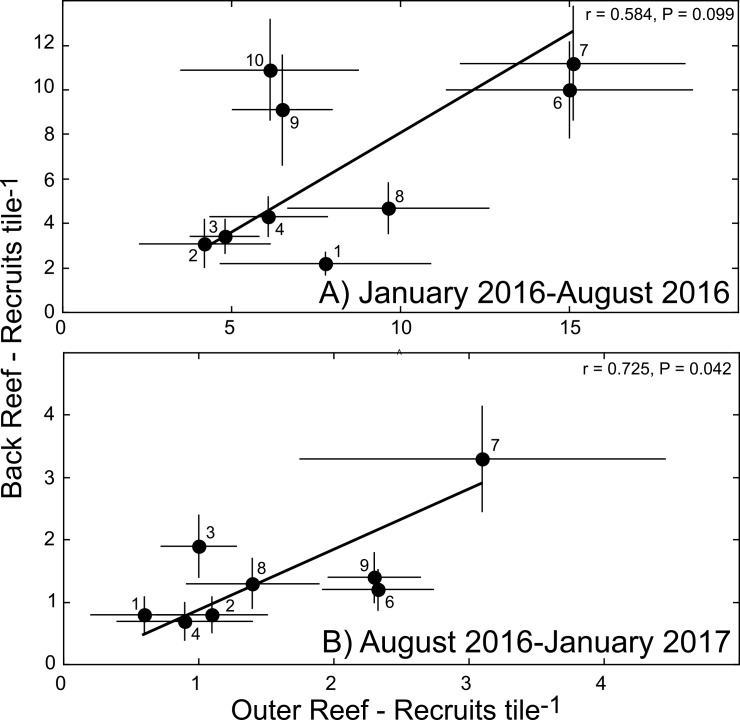
Scatterplots showing mean density of coral recruits (± SE, n = 7–15 tiles site^-1^) on tiles deployed between January 2016 and August 2016 (A) and August 2016 and January 2017 (B) at 8–9 sites with paired deployments on the outer- and back- reef during the El Ni**ñ**o year of 2016. Lines show Model II regressions with GMR slopes [83], and numbers adjacent to symbols indicates sites (as in Fig 1).

**Fig 6:**
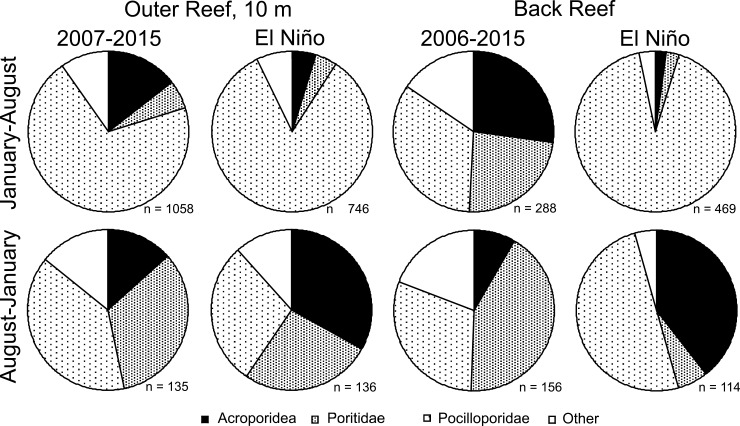
Pie charts showing the taxonomic composition of coral recruits on settlement tiles deployed on the outer- and in the back- reef of Moorea (sites in Fig 1) to sample January-August and August-January of years between 2007–2015 or the El Niño year of 2016. Shading represents proportional contribution of recruits in three families and an unidentified category (“Other”), with the total number of recruits shown for each graph (n).

The majority of coral recruits on the settlement tiles was pocilliporids, acroporids, or poritids in all periods and in both habitats (Fig 5). In the decade prior to 2016, pocilloporids dominated on the outer reef, regardless of sampling period, as well as the back reef from January-August, although poritids were more common from August to January. Poritids were more abundant in the back reef versus the outer reef, and over August-January versus January-August. The taxonomic composition of recruits changed between the initial decade and 2016 on the outer reef between January and August (χ^2^ = 55.112, df = 3, P < 0.001), and August and January (χ^2^ = 14.956, df = 3, P = 0.002), and for the back reef between January and August (χ^2^ = 292.326, df = 3, P < 0.001) and August and January (χ^2^ = 79.550, df = 3, P < 0.001). In 2016 on the outer reef, the relative abundance of pocilloporids increased 19% between January and August, while other taxa became less common, and from August to January, acroporids increased 2.5-fold while other taxa became less common. In 2016 on the back reef, between January and August, pocilloporids accounted for 92% of the recruits (versus 34% in the decade before), and from August to January, the abundance of pocilloporids increased 1.7-fold, and the abundance of acroporids 4.7-fold (Fig 6).

In 2016 when sampling for coral recruitment was conducted at longitudinally paired, back and outer reef sites that were 230–316 m apart (Fig 1), recruitment was positively associated between the two habitats, although the association was only significant over August 2016-January 2017 (r = 0.725, df = 6, P = 0.042), with a trend over January 2016-August 2016 (r = 0.584, df = 7, P = 0.099) (Fig 5). From January to August, the taxonomic composition of the recruits different between habitats (χ^2^ = 18.358, df = 3, P < 0.001), with the effect attributed to small variation in all taxa, but generally, fewer-than-expected acroporids and poritids, and more- than-expected pocilloporids and “others” in the outer versus the back reef (Fig 6). From August to January, the taxonomic composition of the recruits again differed between habitats (χ^2^ = 26.968, df = 3, P < 0.001), but much of the difference was caused by fewer-than-expected poritids in the back- versus the outer- reef, and also more-than-expected pocilloporids in the back- versus the outer- reef (Fig 6).

## Discussion

During 2014, there were signs that a strong El Niño would develop, but eventually it did not occur until 2015 [3], and then became an intense event [54], which raised concerns over its negative impacts on coral reefs [15]. Coral bleaching was recorded in late 2014 in Kaneohe Bay, Hawai’i [55], and in parts of Hong Kong [56], but these events were attributed (in part) to warming associated with the ending of the cool phase of Pacific Decadal Oscillation (PDO). In 2015, El Ni**ñ**o-related bleaching was common in the Indo-Pacific [15], and in 2015/2016, severe bleaching was recorded along the Great Barrier Reef [14]. With widespread reports of coral bleaching in 2015/2016 [14,15,17], and warm seawater around Mo’orea in January and February 2016 (Fig 2), damaging implications of these trends were expected on the reefs of Mo’orea. In contrast, 2016 was not associated with large-scale negative responses on the outer reef coral community. Instead, answers to the three questions addressed by this study reveal potentially beneficial ecological responses in the severe El-Niño year of 2016. First, 2016 was associated with high recruitment of corals with an unusual representation of families, relative to recruitment recorded in the preceding decade. Second, depending on the strength of the stock-recruitment relationship (sensu [57]) for scleractinians in Mo’orea, high recruitment in 2016 has the potential to initiate changes in coral community structure, particularly in the back reef. Finally, coral recruitment that is variable along the reef (i.e., parallel to shore), and positively associated between paired outer- and back- reef sites, supports the role of cross-reef transport of coral larvae in populating the back reef [58,59], and in the capacity of features of the outer reef (e.g., buttresses and sand channels [60]) to promote variable larval delivery to the benthos. Together, these effects probably will determine how the environmental conditions of the 2016 El Ni**ñ**o year will affect the coral community structure of back- and outer- reef habitats in Mo’orea.

The El Niño year of 2016 brought unusual seawater temperatures to Mo’orea, with the year beginning up to 0.7°C warmer than the decadal mean for January and February, and progressing with an intensification of the anomaly to 0.6–0.7°C above the upper 95%CI of the decadal mean in March and April. During this period, the NOAA Degree Heating Weeks (DHW) metric for the Society Archipelago exceeded 4.0, leading to a Level 1 bleaching alert (Fig 2), and coincident with this development, corals started to bleach on the outer reef of Mo’orea (Fig 3). As the seawater around Mo’orea cooled in May 2016 to long-term in situ mean values, corals on the outer reef regained their normal color (Fig 3). Although in situ seawater temperature again was warmer than the long-term mean from September to December 2016 (up to 1.1°C above the 95%CI of the decadal mean), it remained below the local bleaching threshold of 30.0°C (https://coralreefwatch.noaa.gov/) (Fig 2). Compared to the temperatures experienced on the Great Barrier Reef (GBR) and elsewhere in the Indo-Pacific during 2016, seawater warming in Mo’orea during 2016 was minor. For example, 31% of reefs on the GBR experienced 8–16 degree heating weeks (DHW) in 2016 [14], with temperatures as high as 31.9°C at Thursday Island in the Torres Strait [61], on Dongsha Atoll, Taiwan, 40% of corals on the reef flat died in 2015 when seawater temperature exceeded 6°C above normal summertime values [17], and in the Indian Ocean, coral cover at one Maldivian atoll declined 75% between January and September 2016, during which temperatures were 0.4° above the local bleaching threshold (30.9°C [62]). While bleaching on the outer reef of Mo’orea in 2016 was minor (this study), elsewhere in French Polynesia, warming caused serious bleaching in the giant clam *Tridacna maxima* at Reao Atoll, 1,300 km east of Tahiti [63].

The 2016 El Niño did not bring the long periods of high seawater temperature to Mo’orea that proved damaging to corals on the GBR [14], but nonetheless, it created a distinctive thermal record and was associated with scleractinian recruitment that was unique on a decadal time scale. The history of studying the reefs of Mo’orea provides a lengthy context in which the present analysis can be interpreted, since settlement tiles have been deployed around this island from 1990–1993 [64], 2000–present [65,66], and 2006-present (this study). Comparing among different studies in which settlement tile have been deployed is challenging due to varying methodology, and inferring coral recruitment on meter-square areas of complex natural surfaces based on recruits detected on small tiles of artificial materials is of dubious value. Nevertheless, using settlement tiles as a standardized assay for coral recruitment, the aforementioned studies reported coral recruitment from 1990 to 1993 of 19–133 recruits m^-2^ y^-1^ in the back reef, and 17–84 recruits m^-2^ y^-1^ at 8–10-m depth on the north shore outer reef [64]. From 2000–2003, recruitment rates of 103–181 recruits m^-2^ y^-1^were recorded at 12-m depth at Vaipahu on the north shore [65], and over 2004–2005, 423 recruits m^-2^ y^-1^ were recorded at the same site [66]. A larger geographic context to coral recruitment is provided by settlement tiles deployed in Raiatea and Tahiti in 2004–2005 (12-m depth), which revealed recruitment of 204–3,560 recruits m^-2^ y^-1^, and 117–1,078 recruits m^-2^ y^-1^, respectively [66]. When expressed on the same scale, recruitment in Mo’orea in 2016 ranged from 104–357 recruits m^-2^ y^-1^ on the outer reef, and from 59–284 recruits m^-2^ y^-1^ in the back reef. Together, the data compiled above shows that the density of coral recruits on the north shore of Mo’orea in 2016 is the highest that has been recorded in 12 years (Fig 4), and the second highest since records began in 1990. Interestingly, coral recruitment at 12-m depth on the north shore of Mo’orea was higher over 2004–2005 (423 recruits m^-2^ y^-1^ [66]) than in 2016, and 2004–2005 was a weak El Niño year [67] when seawater temperature around Mo’orea was slightly elevated and exceeded the local bleaching threshold for a short period [68].

Understanding the causes of this biological signal (i.e., as in Fig 4) requires consideration of the constraints of associational data (i.e., it cannot reveal cause-and-effect), and the ways in which the thermal perturbations of 2016 could affect coral reproduction and recruitment. First, it is possible that recruitment in 2016 and the thermal regime of this year were unrelated. Support for this possibility comes from the recent history of the outer, north shore of Mo’orea at 10-m depth, on which high coral cover in 2005 (≈ 38%) was destroyed by *Acanthaster planci* and cyclone Oli (February 2010) [47] to leave only ≈ 4% in 2010 [48]. Destruction was followed by strong coral community recovery that returned coral cover to 26% in 2014 [48] (56% at 10-m depth in 2016, averaged between sites), mediated by high coral recruitment in 2011 [48] that was the highest recorded at the present study sites until the current analysis. While most (73%) of the coral on the recovered outer reef on the north shore is *Pocillopora* spp. (e.g., in 2016), recovery extended to the broader coral community as revealed by the similarity of the multivariate community in 2014 and 2005 [48]. Estimates of the fecundity of *Pocillopora* spp. on the north shore of Mo’orea in 2014 suggest they could produce ≈ 4.5 x 10^12^ zygotes annually and, therefore, could “self seed” (sensu [69]) their own populations [49]. Despite this inferred fecundity, recruitment of pocilloporids to tiles in the back- and outer- reef was modest in 2014 and 2015, and while coral cover on the north shore outer reef (10-m depth) increased between 2015 (44% cover) and 2016 (56% cover), recruitment of pocilloporids (and other taxa) in 2016 was disproportionately higher than in 2015. Together, these observations suggest a “business as usual” scenario to explain high recruitment in 2016 as a function of progressive coral community recovery, is a poor fit to the empirical data.

While it is feasible that El Niño might affect coral recruitment in Mo’orea through mechanisms other than temperature—for example, through depressed sea level [1,70] that could limit cross-reef transport of seawater and the larvae it contains [58,59]—without data supporting this hypothesis, it is less parsimonious than mechanisms relying on temperature. Unfortunately, it is not possible to retrospectively evaluate coral fecundity without archived samples (which are not available for Mo’orea) and, therefore, it is not possible to evaluate the role of variable fecundity in driving the differences in recruitment reported in the present study. However, assuming that coral fecundity at least was not greater in 2016 versus 2014 or 2015, which is reasonable given the impairment of reproduction that might be expected from thermal bleaching [34,35], then high coral recruitment in 2016 may reflect an effect of temperature on the timing of spawning, fertilization and the development of pelagic larvae, or the growth of the recruits they produce. Seawater temperature has long been known to affect the phenology of coral spawning [37,71], with warm temperatures advancing release relative to cool temperatures [37, 51], and recently this relationship has been quantified for two brooding pocilloporids in Taiwan [37]. For these corals, the mean lunar day of release of brooded larvae advanced with rising temperature, and would advance 12–15% (≈ 1 d) with an increase from 28°C to 29°C [37]. If similar effects occur with the spawning corals in Mo’orea, then the warm temperatures in 2016 might have advanced spawning to a similar extent. For spawning advanced by ≈ 1 d to affect recruitment, then advance-release gametes would have to encounter hydrodynamic regimes promoting variation in larval dispersal over a few days. The consistency of water flow and wave height on the north shore of Mo’orea suggests this is unlikely [58].

The most likely means by which elevated temperature could have mediated recruitment in Mo’orea is through effects on pelagic larval durations (PLD) [72], and calcification [73] of coral recruits. The PLD of a wide variety of marine organisms is inversely temperature-dependent [72], but the population-averaged response for 72 species is small (≈ 5%) for an increase from 29°C to 31°C (Fig 5A in [72]). Such an effect size applied to larvae of tropical corals is unlikely to be ecologically meaningful for Mo’orea, because it amounts to ≈ 0.5 d difference in the PLD of broadcast spawners [74]. If the PLD of coral larvae in Mo’orea was shortened by ≈ 0.5 d, this is unlikely to affect dispersal between the outer- and back- reef habitats, between which seawater repeatedly exchanges at ≈ 20 cm s^-1^ [58]. However, coral larvae probably are more sensitive to temperature than most of the organisms considered by O’Connor et al. [72], and indeed, short-term experimental work shows that coral larvae settle more quickly at elevated to lower (control) temperatures [52,75,76]. For example, settlement of the larvae of *Acropora solitaryensis* increased 242% following 1 d exposure to 29° versus 26°C in experiments conducted at Lord Howe Islands, southern GBR [75]. With an effect size of comparable magnitude in Mo’orea, an increase of ≈ 1°C in seawater temperature could accentuate settlement and contribute to the recruitment signal reported herein.

Finally, it is possible that the environmental conditions of 2016 contributed to rapid growth of coral recruits, which can serve as a mechanism to avoid the high risks of remaining small [77], thus accentuating the size of the recruiting cohort. Although growth rates of recruits were not recorded, a large number with numerous corallites was found on the tiles in September 2016 (e.g., 7.6 ± 0.5 corallites recruit^-1^ [mean ± SE, n = 162] for the back reef) relative to previous years (when most had 1–3 corallites recruit^-1^ [P.J. Edmunds personal observation]). This trend suggests that growth rates of recruits in 2016 may have been more rapid than in the previous decade. Seawater temperature again is a likely cause of this effect, as temperature accentuates coral growth depending on the range over which the increase occurs relative to the thermal threshold for maximum growth [73].

**Fig 7:**
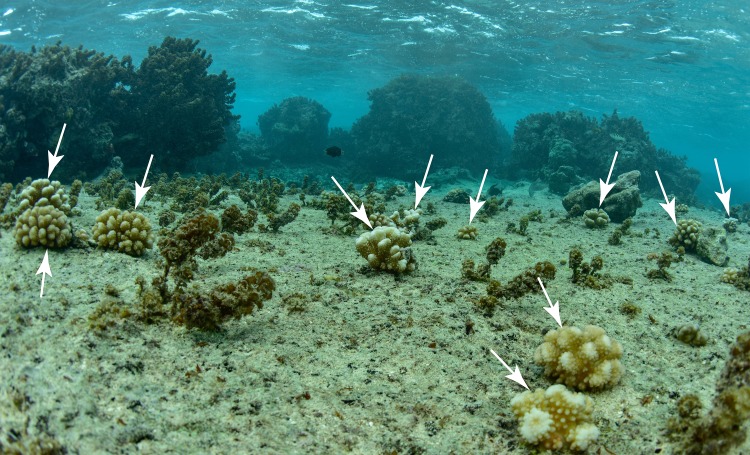
Photograph of the Mo’orea back reef, ≈ 50-m south of the reef crest, to the west of site 6 (Fig 1) in May 2017. The water is about 2-m depth and on the smooth carbonate pavement a large number of juvenile corals in the 4–10 cm size class can be found (arrows), most of which are *Pocillopora*, although *Acropora* and *Porites* also can be found.

It is too soon to determine whether coral recruitment in 2016 in Mo’orea will promote a change in coral community structure, but if survivorship of the recruits is sufficiently high, such an outcome is feasible given the high density, and the taxonomic composition of the recruits. The taxonomic effect is striking in the back reef, where 2016 brought strong representation of pocilloporids from January to August (2% of all recruits), and many acroporids and pocilloporids from August 2016 to January 2017 (40% and 50%, respectively, of recruits). Realizing the possibility of change in coral community structure for the outer reef at 10-m depth on the north shore seems unlikely, however, because most of the benthos (56%) was covered by coral in 2016, and the majority was *Pocillopora* spp. The back reef on the north shore provides more opportunities for recruitment to modify coral community structure, because large areas of hard substrata were available in 2016 (34 ± 4%, mean ± SE [n = 10] was crustose coralline algae or bare space), and the availability of these substratum categories have been accentuated by coral mortality that has reduced coral cover on the north shore from 29.1 ± 11.4% in 2005 to 4.8 ± 2.4% in 2016 (both ± SD, n = 2), and on a longer time scale, has depleted the community of *Acropora* spp. [78–80]; *Acropora* spp. cover on the north shore back reef in 2015 was ≈ 0.05%. Macroalgae, particularly *Turbinaria* and *Sargassum*, also are becoming more abundant in the back reef of Moorea [81], particularly on the north shore (20 ± 4% in 2016, mean ± SE [n = 10]), where they are likely to deter coral settlement [82]. Thus, while the present study shows high coral recruitment in the back reef in 2016, and evidence of elevated relative abundance of acroporids from August 2016 to January 2017, whether these trends portend changes in coral community structure is likely to depend on the extent to which the survival if these coral recruits is affected by macroalgae. Large numbers of juvenile corals on reef pavement just behind the reef crest in May 2017 suggest a change in benthic community structure may already be underway (Fig 7).

The results of this study, together with recently-published results [14], emphasize that the impacts of the 2016 El Niño on coral reefs were inconsistent across the Indo-Pacific. One reason for this discrepancy is that severe and persistent seawater warming did not extend to Mo’orea in 2016, whereas it was striking on the Great Barrier Reef [14], but this explanation cannot account for the biological signal registered in the recruiting coral population of 2016 in Mo’orea. While it is not possible to reject the hypothesis that the high coral recruitment of 2016 reflects the recovery of these coral communities since 2010 [47–49], and is independent of seawater temperature, this possibility is inconsistent with the apparent threshold effect for coral recruitment as 2015 transitioned to 2016. Instead, the recent ecological history of these reefs may have primed them for sensitivity of coral recruitment to sub-lethal, upward excursions of seawater temperature. The variation in coral recruitment along the north shore of Mo’orea, and the offshore-onshore association of recruitment in adjacent habitats (outer reef and back reef, Fig 5), reveals spatial structuring in recruitment that may contribute to spatial variation in coral communities; the next few years will reveal whether this possibility is realized in the back reef. Generalizations regarding negative effects of the 2015/16 El Niño year on coral reefs in the Indo-Pacific appear incorrect for Mo’orea, and reefs in this location continue to exhibit features characteristic of high ecological resilience [49].
